# Editorial: The immune infiltrate as a paradigm model to study the biology and novel therapeutic approaches in sarcomas

**DOI:** 10.3389/fendo.2023.1334519

**Published:** 2023-12-04

**Authors:** Federica Recine, Alberto Bongiovanni, Laura Mercatali, Valentina Fausti, Virginia Ferraresi, Alessandro De Vita

**Affiliations:** ^1^ Medical Oncology Unit, Azienda Ospedaliera “San Giovanni Addolorata”, Roma, Italy; ^2^ Clinical and Experimental Oncology, Immunotherapy, Rare Cancers and Biological Resource Center, IRCCS Istituto Romagnolo per lo Studio dei Tumori (IRST) “Dino Amadori”, Meldola, Italy; ^3^ Osteoncology, Bone and Soft Tissue Sarcomas and Innovative Therapies Unit, IRCCS Istituto Ortopedico Rizzoli, Bologna, Italy; ^4^ Sarcomas and Rare Tumors Unit, Istituti di Ricovero e Cura a Carattere Scientifico (IRCCS) Regina Elena National Cancer Institute, Rome, Italy; ^5^ Preclinic and Osteoncology Unit, Biosciences Laboratory, IRCCS Istituto Romagnolo per lo Studio dei Tumori (IRST) “Dino Amadori”, Meldola, Italy

**Keywords:** sarcomas, immunotherapy, tumor microenvironment, extracellular matrix, macrophages, T-cell infiltration

The tumor immune microenvironment (TiME) plays an essential role in tumor development as well as in tumor metastatization in a plethora of neoplasms, including soft tissue sarcoma (STS) and bone sarcomas (1). STS and bone sarcomas are a heterogeneous group of solid malignancies, encompassing more than 100 histological subtypes, with different biological and clinical behaviors, genomic backgrounds, and responses to treatments (2–4).

TiME is a dynamic milieu of various types of elements, such as infiltrating immune cells, tumoral cells, and extracellular matrix, that have a potential role in the cancer growth process. Further understanding of the TiME mechanism is crucial for the development of new therapeutic agents, such as immunotherapy and its clinical application. Moreover, studying the role of the TiME in STS may contribute to improving the knowledge of the natural history and disease biology in this heterogenous group of rare mesenchymal tumors (5, 6).

Articles published as part of the Research Topic “*The Immune Infiltrate as a Paradigm Model to Study the Biology and Novel Therapeutic Approaches in Sarcomas*” highlight the recent discoveries in this field, emphasizing the crucial role of the TiME in the etiopathogenesis of STS with the correlated clinical implications.

Wu et al. explored the correlation between angiogenesis and the TiME in osteosarcoma (OS) that underlies the network between the vessel state and immune infiltrate in this neoplasm. In the study, two subgroups of OS patients were identified according to the expression of angiogenesis-related genes (ARGs), and the vessel state and TiME of these groups were compared. The ARG score showed the correlation with angiogenesis and characterized the immune landscape of OS, leading to a risk score model and a prediction model of OS in the prognosis and response to immunotherapy.

In this context, Liao et al. focused on the role of sulfatinib, a novel multi-targeted tyrosine kinase inhibitor (TKI), in the management of OS patients. This agent showed dual activity as a multi-target TKI that inhibits proliferation in OS cells, by phosphorylating FGFR1 and downstream kinases, and modulates the TiME, increasing chemotherapy sensitivity.

Furthermore, in bone sarcomas, Weil and Loeb provided a detailed overview of the bone tumor microenvironment with a description of the various potential cancer mechanisms in this interplay. The authors emphasized the role of the crosstalk between sarcomas and the bone microenvironment, contributing to the control of tumor growth and cell extravasation and metastasis, as well as the implicated therapeutic potential options.

Another sarcoma histotype in which there is a relationship between systemic immune activation, angiogenesis, and tumor pathogenesis is Kaposi’s sarcoma (KS). The authors reported a potential correlation between soluble markers of HIV-1-related immune activation and the level of growth factors in HHV-8 seropositive KS patients (Nana et al.).

The crucial role of TiME in tumorigenesis was also evaluated in epithelial tumors; in particular, in lung cancer, as described by (Zhao et al.). The authors hypothesized a correlation between the changes in serum cytokine levels and the effectiveness of targeted immunotherapy in lung cancer and suggested immunological biomarkers as prognostic predictors for lung cancer.

In conclusion, the role of the TiME in the pathogenesis of tumors is still being studied due to the complexity and heterogeneity of this highly dynamic network of various elements. In this context, targeting the tumor microenvironment may be a promising therapeutic strategy for reducing tumor growth and metastasis and blocking therapeutic resistance.

## Author contributions

FR: Writing – original draft. AB: Writing – review & editing. LM: Writing – review & editing. VFa: Writing – review & editing. VFe: Writing – review & editing. ADV: Writing – review & editing.
